# Severe Adverse Maternal Outcomes among Women in Midwife-Led versus Obstetrician-Led Care at the Onset of Labour in the Netherlands: A Nationwide Cohort Study

**DOI:** 10.1371/journal.pone.0126266

**Published:** 2015-05-11

**Authors:** Ank de Jonge, Jeanette A. J. M. Mesman, Judith Manniën, Joost J. Zwart, Simone E. Buitendijk, Jos van Roosmalen, Jeroen van Dillen

**Affiliations:** 1 Department of Midwifery Science, AVAG and the EMGO Institute of Health and Care Research, VU University Medical Center, Amsterdam, the Netherlands; 2 Leiden University Medical Center, Department of Obstetrics, Leiden, the Netherlands; 3 Deventer Hospital, Department of Obstetrics, Deventer, the Netherlands; 4 Leiden University Medical Center, Leiden, the Netherlands; 5 Athena Institute, VU University Medical Center, Amsterdam, the Netherlands; 6 Radboud University Medical Center Nijmegen, Department of Obstetrics, Nijmegen, the Netherlands; University of Barcelona, SPAIN

## Abstract

**Objective:**

To test the hypothesis that it is possible to select a group of low risk women who can start labour in midwife-led care without having increased rates of severe adverse maternal outcomes compared to women who start labour in secondary care.

**Design and Methods:**

We conducted a nationwide cohort study in the Netherlands, using data from 223 739 women with a singleton pregnancy between 37 and 42 weeks gestation without a previous caesarean section, with spontaneous onset of labour and a child in cephalic presentation. Information on all cases of severe acute maternal morbidity collected by the national study into ethnic determinants of maternal morbidity in the Netherlands (LEMMoN study), 1 August 2004 to 1 August 2006, was merged with data from the Netherlands Perinatal Registry of all births occurring during the same period.

Our primary outcome was severe acute maternal morbidity (SAMM, i.e. admission to an intensive care unit, uterine rupture, eclampsia or severe HELLP, major obstetric haemorrhage, and other serious events). Secondary outcomes were postpartum haemorrhage and manual removal of placenta.

**Results:**

Nulliparous and parous women who started labour in midwife-led care had lower rates of SAMM, postpartum haemorrhage and manual removal of placenta compared to women who started labour in secondary care. For SAMM the adjusted odds ratio’s and 95% confidence intervals were for nulliparous women: 0.57 (0.45 to 0.71) and for parous women 0.47 (0.36 to 0.62).

**Conclusions:**

Our results suggest that it is possible to identify a group of women at low risk of obstetric complications who may benefit from midwife-led care. Women can be reassured that we found no evidence that midwife-led care at the onset of labour is unsafe for women in a maternity care system with a well developed risk selection and referral system.

## Introduction

In many Western countries obstetricians are medically responsible for birth, even if midwives or obstetric nurses provide intrapartum care.[1] In some maternity care models, however, midwives are responsible for birth as well. In the Netherlands, 85% of women start pregnancy in midwife-led care of whom 41% are referred to obstetrician-led care in hospital.[2] Of all women, 51% start labour in midwife-led care and they can choose to give birth at home or in hospital.[2] For convenience, we will use the term ‘midwife-led care’ for all women who started labour in primary care, either under the responsibility of midwives (more than 99%) or of general practitioners (less than 1%). If risk factors or complications occur, as described in the ‘Obstetric Indication List’, women are referred to obstetrician-led care for which obstetricians are responsible although clinical midwives may be providing most of the care.[3,4] Examples of indications for referral during pregnancy are: previous caesarean section, hypertension, induction of labour and post term gestation [5]. In addition, some low risk women will choose to be in obstetrician-led care without being referred by a midwife, for example if they have a history of subfertility and are therefore already familiar with the obstetric unit. Sense of safety is the most important argument for low risk women to opt for obstetrician-led care in the Netherlands.[6] In one Dutch study, it was estimated that about 18% of all *low risk* women start labour in obstetrician-led care.[7] Of all pregnant women, 30% give birth in midwife-led care.[2]

In previous studies, we found no increased risk of adverse perinatal or maternal outcomes among planned home versus planned hospital births for women in midwife-led care at the onset of labour.[4,8] However, these studies did not answer the question whether start of labour in midwife-led care, regardless of planned place of birth, leads to a higher rate of adverse outcomes compared to start of labour in obstetrician-led care.

Concerning perinatal outcomes, one Dutch observational study showed better outcomes among women who started labour in obstetrician-led versus midwife-led care [9], but the interpretation of these results is subject to debate because of methodological shortcomings in the study design.[10,11] For example, all cases of intrapartum and neonatal mortality were included from midwifery practices and hospitals in one neonatal intensive care unit (NICU) region even though some of these babies were born to women in neighbouring regions. However, for the denominator all births were included from women who lived in the index NICU region only. Particularly for midwifery practices on the border of this NICU region, this may have inflated their intrapartum and neonatal mortality rates.[10] Even so, the study has cast doubt on the safety of the Dutch maternity care system.[12] International studies comparing midwife-led models of care with models in which medical doctors are responsible or shared care is provided have shown similar or lower risks of postpartum haemorrhage [1,13,14] and blood transfusion [15] among women in midwife-led care. However, these studies were not large enough to examine rare but severe adverse maternal outcomes. In the Netherlands, primary care midwives are the lead professionals for low risk women. Referral from midwife-led to obstetrician-led care during labour may cause delay and vital information could be lost during handover. This might increase the risk of maternal morbidity, especially in acute situations. Nevertheless, we hypothesized that it is possible to select a group of low risk women who can start labour in midwife-led care without having increased rates of severe adverse maternal outcomes compared to women who start labour in obstetrician-led care.

We used merged data from two large Dutch national datasets which enabled us to examine rare outcomes. The aim of the study was to compare the rates of severe acute maternal morbidity (SAMM), postpartum haemorrhage and manual removal of placenta among births that started in midwife-led care versus those that started in obstetrician-led care.

## Materials and Methods

The Dutch maternity care system and linkage of the two national datasets that we used have been described elsewhere.[4] In short; data were combined from the ‘Nationwide study into Ethnic Determinants of Maternal Morbidity in the Netherlands (the LEMMoN study) and from the Netherlands Perinatal Registry (PRN).[4]

### Ethics statement

The ethical committee of VU University Medical Center confirmed that ethical approval is not necessary for this cohort study (reference number 11/399).

### Study sample

We selected women with a singleton pregnancy without a known history of caesarean section who gave birth between 37 and 42 weeks of gestation and who had spontaneous onset of labour. From the LEMMoN dataset, we only included women who had SAMM after onset of labour from 37 to 42 weeks gestation. Women who had SAMM during pregnancy, but recovered and gave birth at term, were excluded.

The PRN database consists of data from three separate databases: one for primary midwife-led care (national perinatal database-1), one for secondary obstetrician-led care (national perinatal database-2), and one for paediatric care (national neonatal database). General practitioners who still attend births and a few primary care midwives do not register their data in the national register and their National Perinatal Database-1 forms will be missing. Their uncomplicated primary care births are therefore not registered. However, if complications arise, their births will be registered via the national perinatal database-2. To prevent selection bias, we excluded women who were referred during or after labour from midwife-led to obstetrician-led care but for whom the National Perinatal Database-1 form was missing.

We also excluded women who gave birth to a child in non-cephalic or unknown presentation or for whom planned level of care at the onset of labour was not known.

Hence, the study sample consisted of women with term singleton pregnancy without a known history of caesarean section, with spontaneous onset of labour and a child in cephalic presentation.

### Definition of variables

The main outcome variable was overall SAMM, which was recorded in the LEMMoN dataset. SAMM comprised five different categories which were also examined separately as secondary outcomes: admission to intensive care, uterine rupture, eclampsia or HELLP with liver haematoma or rupture, major obstetric haemorrhage (blood transfusion of four or more packed cells) and other SAMM as diagnosed by the attending clinician, such as pulmonary or amniotic fluid embolism. Other secondary outcomes were postpartum haemorrhage (defined as at least 1000 ml of blood loss) and manual removal of placenta. These outcomes were recorded in the PRN dataset. Women could have more than one adverse outcome. For example, all women who had major obstetric haemorrhage were also classified as having had postpartum haemorrhage. However, women with SAMM were only counted once for this outcome, even if they had complications in two SAMM categories; for example if they had a major obstetric haemorrhage and were admitted to intensive care.

We identified characteristics that have previously been associated with level of care at the start of labour and maternal complications: parity, maternal age, ethnic background and socioeconomic position.[7,9,16–18] Previously, gestational age was found to be associated with adverse maternal outcomes and with planned place of birth and we assumed it might be related to level of care at the onset of labour as well.[4,17] Gestational age was divided into 37 to 37+6 weeks, 38 to 40+6 weeks and 41+0 to 41+6 weeks gestation. Parity was coded as nulliparous or parous. Maternal age was coded as below 25 years, between 25 and 34 years and 35 years or older. Ethnic background was categorised as ‘Dutch’ and ‘non-Dutch’.[4] Socioeconomic position was derived from scores based on postal codes developed by the National Institute for Social Research (SCP; ‘Sociaal Cultureel Planbureau’). These scores were divided into low, medium and high based on the P25 and P75 cut-off points.

In a secondary analysis, we controlled for augmentation of labour with oxytocin and operative delivery (caesarean section, vacuum or forceps delivery), both as binary variables, because these have been found to be associated with adverse maternal outcomes.[16,17,19]

### Data analyses

For births that started in midwife-led and obstetrician-led care, the numbers and percentages of the primary and secondary outcomes were calculated. Logistic regression analyses were carried out for nulliparous and parous women separately to compare rates of adverse maternal outcomes among women who started in midwife-led versus in obstetrician-led care; these were only performed for SAMM, major obstetric haemorrhage, postpartum haemorrhage and manual removal of placenta, because the other outcomes were too rare to be able to control the analyses for women’s characteristics. The crude odds ratio’s and 95% confidence intervals were calculated and multivariable logistic regression analyses were used to control for potential confounders (i.e. maternal age, ethnic background, socioeconomic position and gestational age at delivery), resulting in adjusted odds ratio’s with 95% confidence intervals. Cases with missing data were excluded.

Subsequently, the associations between planned level of care and SAMM were controlled for augmentation of labour with oxytocin and operative delivery to examine whether these factors could explain possible differences found.

Level of care at the onset of labour is based on information from the national perinatal database-1 and national perinatal database -2. For the main analyses, we used the official definition from the PRN. Sensitivity analyses were conducted for different definitions of level of care at the onset of labour; for women without discrepancies between national perinatal database-1 and national perinatal database-2 and for women with onset of labour based on the national perinatal database-1 only. Finally, we repeated the analyses without women in obstetrician-led care at the onset of labour who were referred from midwife-led to obstetrician-led care during pregnancy or for whom a risk factor was recorded which would have been a reason for antenatal referral, had they been in midwife-led care.

## Results

### Study population

**Fig 1:**
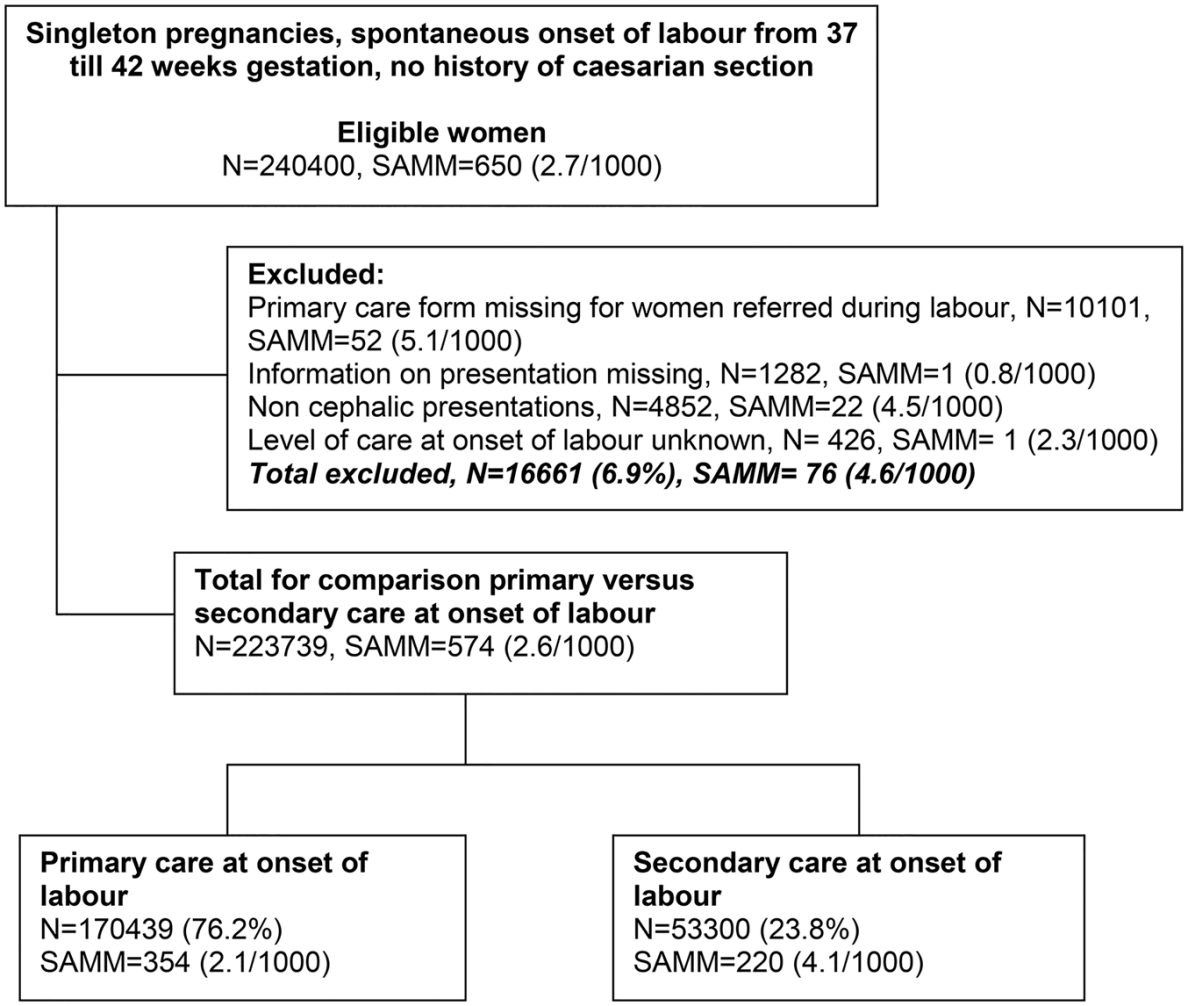
Flow chart: births between August 2004 and July 2006.

Of the total linked data, 240 400 women had a spontaneous onset of labour between 37 and 42 weeks gestation and no known history of caesarean section (Fig 1). In the LEMMoN study, all women in the eligible group with a previous caesarean section were identified from the case notes; 27.4% of these women had no record of a previous caesarean section in the PRN which illustrates that this variable is underreported in the routine registration data. From 10 101 women referred during or after labour the national perinatal database-1 form was missing and of these 52 had SAMM. Information on presentation was missing for babies born to 1282 women (SAMM, n = 1) and another 4852 babies were born in non-cephalic presentations (SAMM, n = 22). For 426 women level of care at the onset of labour was unknown (SAMM, n = 1). Of the remaining 223 739 women, 170 439 started labour in midwife-led care and 53 300 in obstetrician-led care.

Women in midwife-led care at the onset of labour were more likely to be parous, less likely to give birth between 37+0 and 37+6 weeks gestation; they were less often 35 years or older; they were more often of Dutch origin and less often had a lower socioeconomic position (Table 1).

**Table 1:** Characteristics of women in midwife-led and obstetrician-led care at the onset of labour with a singleton pregnancy, cephalic presentation, no history of caesarean section, spontaneous onset of labour between 37 and 42 weeks.

		Total	Midwife-led care at onset of labour	Obstetrician-led care at onset of labour
		N = 223,739 N (%)	N = 170,439 N (%)	N = 53,300 N (%)
**Parity** ^**1**^	Para 0	100,686 (45.0)	76,435 (44.8)	24,251 (45.5)
	Para 1+	123,044 (55.0)	93,995 (55.2)	29,049 (54.5)
**Gestational age** ^**2**^	37+0 to 37+6	11,034 (4.9)	7,011 (4.1)	4,023 (7.5)
	38+0 to 40 +6	163,750 (73.2)	125,359 (73.6)	38,391 (72.0)
	41+0 to 41 + 6	48,955 (21.9)	38,069 (22.3)	10,886 (20.4)
**Maternal age** ^**2**^	< 25 years	28,062 (12.5)	21,695 (12.7)	6,367 (11.9)
	25 to 34 years	150,735 (67.4)	117,717 (69.1)	33,018 (62.0)
	≥ 35 years	44,921 (20.1)	31,011 (18.2)	13,910 (26.1)
**Ethnic background** ^**2**^	Dutch	179,656 (80.8)	138,493 (81.8)	41,163 (77.6)
	Non-Dutch	42,812 (19.2)	30,911 (18.2)	11,901 (22.4)
**Socioeconomic position** ^**2**^	High	52,904 (24.0)	40,804 (24.3)	12,100 (23.2)
	Medium99	99,758 (45.3)	77,303 (46.0)	22,455 (43.0)
	Low	67,765 (30.7)	50,070 (29.8)	17,695 (33.9)

Missing values: parity N = 9 (0%), maternal age N = 21 (0%), ethnic background N = 1271 (0.6%), Socioeconomic position N = 3312 (1.5%)

^1^ P<0.05

^2^ P<0.001

Fewer women who started labour in midwife-led versus in obstetrician-led care received augmentation of labour (nulliparous women 27.4% versus 43.5% and parous women 6.1% versus 23.0% respectively) or had an operative delivery (nulliparous women 24.0% versus 36.5% and parous women 2.2% versus 11.5%) (Table 2).

**Table 2:** Medical interventions among nulliparous and parous women who started labour in midwife-led and obstetrician-led care.

	Nulliparous women, N = 100,686		Multiparous women, N = 123,044	
	Midwife-led care at onset of labour	Obstetrician-led care at onset of labour	Midwife-led care at onset of labour	Obstetrician-led care at onset of labour
	N = 76,435	N = 24,251	N = 93,995	N = 29,049
Augmentation of labour N(%)	20,943 (27.4)	10,544 (43.5)	5,739 (6.1)	6,684 (23.0)
Operative delivery N(%)*	18,366 (24.0)	8,841 (36.5)	2,049 (2.2)	3,347 (11.5)

*Operative delivery: caesarean section, vacuum or forceps delivery

### Adverse maternal outcomes

Of all women included in the study, 574 (2.6 per 1000) had SAMM (Table 3). Among women in midwife-led care at the onset of labour, 354 women had SAMM (2.1 per 1000) and among women in obstetrician-led care 220 women had SAMM (4.1 per 1000). Most women who had SAMM had a major obstetric haemorrhage. Postpartum haemorrhage was the most common adverse maternal outcome occurring in 6004 women in midwife-led care (35.3 per 1000) and in 2977 women in obstetrician-led care (59.1 per 1000).

**Table 3:** Severe acute maternal morbidity (SAMM), postpartum haemorrhage and manual removal of placenta among women who started labour in midwife-led and obstetrician-led care.

	Total	Midwife-led care at onset of labour	Obstetrician-led care at onset of labour
	N = 223,739	N = 170,439	N = 53,300
**Severe acute maternal morbidity, N (N/1000)**	574 (2.6)	354 (2.1)	220 (4.1)
*Admission to ICU*, N (N/1000)	151 (0.7)	82 (0.5)	69 (1.3)
*Uterine rupture*, *N (N/1000)*	8 (0)	4 (0)	4 (0.1)
*Eclampsia or severe HELLP syndrome*, N (N/1000)	36 (0.2)	23 (0.1)	13 (0.2)
*Miscellaneous*, N (N/1000)	29 (0.1)	14 (0.1)	15 (0.3)
*Major obstetric haemorrhage (blood transfusion ≥ 4 p*.*c)*, N (N/1000)	501 (2.2)	312 (1.8)	189 (3.5)
*Maternal death* N (N/1000)	9 (0)	5 (0)	4 (0.1)
**Postpartum haemorrhage > 1000mls, N (N/1000)**	8,981 (40.7)	6,004 (35.3)	2,977 (59.1)
**Manual removal of placenta, N (N/1000)**	6,521 (29.9)	3,671 (21.9)	2,850 (56.9)

HELLP = haemolysis, elevated liver enzymes and low platelet count

Missing values: postpartum haemorrhage N = 3213 (1.4%), manual removal of placenta N = 5927 (2.6%)

Women could have more than one type of adverse outcome.

Adverse outcomes were less common among women in midwife-led care at the onset of labour compared to those in obstetrician-led care for nulliparous and parous women (Table 4).

**Table 4:** Severe acute maternal morbidity, postpartum haemorrhage and manual removal of placenta among nulliparous and parous women.

		Nulliparous women N = 100,686		Parous women N = 123,044	
		Midwife-led care at onset of labour	Obstetrician-led care at onset of labour	Midwife-led care at onset of labour	Obstetrician-led care at onset of labour
		N = 76,435	N = 24,251	N = 93,995	N = 29,049
**Severe acute maternal morbidity**	N (N/1000)	215 (2.8)	127 (5.2)	139 (1.5)	93 (3.2)
	Crude OR (95% CI)	0.54 (0.43, 0.67)	Reference	0.46 (0.36, 0.60)	Reference
	Model 1, adj OR (95% CI)*	0.57 (0.45, 0.71)	Reference	0.47 (0.36, 0.62)	Reference
	Model 2, adj OR (95% CI)^¥^	0.64 (0.51, 0.80)	Reference	0.60 (0.45, 0.80)	Reference
**Major obstetric haemorrhage (Blood transfusion ≥ 4 p.c.)**	N (N/1000)	190 (2.5)	110 (4.5)	122 (1.3)	79 (2.7)
	Crude OR (95% CI)	0.55 (0.43, 0.69)	Reference	0.48 (0.36, 0.63)	Reference
	Model 1, adj OR (95% CI)*	0.57 (0.45, 0.73)	Reference	0.48 (0.36, 0.64)	Reference
	Model 2, adj OR (95% CI)^¥^	0.63 (0.49, 0.80)	Reference	0.58 (0.43, 0.79)	Reference
**Postpartum haemorrhage**	N (N/1000)	3389 (44.4)	1449 (62.7)	2615 (27.9)	1528 (56.0)
	Crude OR (95% CI)	0.70 (0.65, 0.74)	Reference	0.48 (0.45, 0.52)	Reference
	Model 1, adj OR (95% CI)*	0.70 (0.66, 0.75)	Reference	0.48 (0.45, 0.52)	Reference
	Model 2, adj OR (95% CI)^¥^	0.72 (0.68, 0.77)	Reference	0.52 (0.49, 0.56)	Reference
**Manual removal of placenta**	N (N/1000)	2347 (31.5)	1375 (60.0)	1324 (14.2)	1475 (54.3)
	Crude OR (95% CI)	0.51 (0.48, 0.55)	Reference	0.25 (0.23, 0.27)	Reference
	Model 1, adj OR (95% CI)*	0.52 (0.48, 0.56)	Reference	0.25 (0.23, 0.27)	Reference
	Model 2, adj OR (95% CI)^¥^	0.57 (0.53, 0.62)	Reference	0.29 (0.27, 0.31)	Reference

*Model 1: adjusted for gestational age, maternal age, ethnic background, socioeconomic position.

^¥^Model 2: adjusted for gestational age, maternal age, ethnic background, socioeconomic position, augmentation of labour and operative delivery.

Missing values: see Tables 1 and 3.

Among nulliparous women the adjusted odds ratio’s and 95% confidence intervals for midwife-led versus obstetrician-led care were: SAMM, 0.57 (0.45 to 0.71), major obstetric haemorrhage, 0.57 (0.45 to 0.73), postpartum haemorrhage, 0.70 (0.66 to 0.75), and manual removal of placenta, 0.52 (0.48 to 0.56). Among parous women outcomes for midwife-led versus obstetrician-led care were: SAMM 0.47 (0.36 to 0.62), major obstetric haemorrhage, 0.48 (0.36 to 0.64), postpartum haemorrhage, 0.48 (0.45 to 0.52), and manual removal of placenta, 0.25 (0.23 to 0.27). When the results were controlled for augmentation of labour and operative delivery, the adjusted odds ratio’s increased. For example, when comparison of SAMM in midwife-led versus obstetrician-led care was controlled for augmentation of labour and operative delivery, the adjusted odds ratio increased from 0.57 to 0.64 (0.51 to 0.80) for nulliparous women. For parous women, the adjusted odds ratio for SAMM increased from 0.47 to 0.60 (0.45 to 0.80) after controlling for these interventions.

In total, nine women died. Of the five women who were in midwife-led care at the onset of labour, four had been referred to obstetrician-led care during labour because of meconium stained liquor. Three of them died some days or weeks after they had been discharged from hospital in good condition; one had thrombosis and two died of causes unrelated to childbirth. The other woman who was referred died of sudden collapse during labour and no cause of death was found at post-mortem examination. One woman gave birth at home and was admitted to hospital after birth where she died the next day of the consequences of eclampsia. Of the four women who were in obstetrician-led care at the onset of labour, two died of severe postpartum haemorrhage and one of eclampsia. One woman had thrombosis more than a week after birth, after she had been discharged home in good condition.

### Sensitivity analyses

Sensitivity analyses for different definitions of level of care at the onset of labour (respectively based on a sample with no discrepancies between national perinatal database-1 and -2 and based on a definition using national perinatal database-1 only) showed similar results for all outcomes (S1 Table). Equally, after excluding women who started labour in obstetrician-led care and who were referred from midwife-led to obstetrician-led care during pregnancy or who had a risk factor recorded in pregnancy, results were similar; for SAMM the adjusted odds ratio’s and 95% confidence intervals for midwife-led versus obstetrician-led care were among nulliparous women 0.60 (0.42 to 0.85) and among parous women 0.47 (0.34 to 0.66).

## Discussion

### Main findings

To our knowledge, this is the largest study so far comparing severe adverse maternal outcomes among women in midwife-led versus obstetrician-led care at the onset of labour. Nulliparous and parous women who started labour in midwife-led care had lower rates of SAMM, postpartum haemorrhage and manual removal of placenta compared to women who started labour in obstetrician-led care. The differences were reduced slightly when results were adjusted for medical interventions and more so for parous than for nulliparous women.

### Strengths and limitations

The main strength of our study is the large sample size. All hospitals in the Netherlands collected cases of SAMM over a period of two years.

Our study has some limitations which we described in our earlier paper.[4] One limitation concerns a degree of uncertainty regarding the level of care at the onset of labour. However, sensitivity analyses using different definitions of the variable level of care at the start of labour generated similar results. Secondly, the data were collected from 2004 to 2006 but we have no reason to believe that changes in women’s characteristics or labour management would lead to more unfavourable outcomes for women in midwife-led care at present.[4] Ethnicity was very broadly defined as ‘Dutch’ and ‘non-Dutch’ because of limitations in the registration data; this definition poorly reflects the diversity of the Dutch population.

Risk factors can be recorded in the national perinatal database-2, but these are not obligatory items and therefore not always registered. For example, we found that a quarter of women with a previous caesarean section according to the information in the LEMMoN study, had no record of this in PRN. An association between previous caesarean section and SAMM has been demonstrated earlier.[19] We excluded all cases of SAMM with a history of caesarean section because this was registered in the LEMMoN database. This means we took more women with a history of caesarean section out of the numerator than the denominator and this may have decreased the rate of SAMM somewhat in the obstetrician-led care group. For postpartum haemorrhage and manual removal of placenta, the results may have been confounded by higher risks among women in obstetrician-led care with an unrecorded history of caesarean section. This may explain why differences between the study groups were much larger for parous compared to nulliparous women for these outcomes but far less so for our primary outcome SAMM.

Other risk factors may be present in the obstetrician-led care group that we did not identify. Controlling for known risk factors only had a very small effect which suggests that other risk factors, in addition to previous caesarean section, are not always recorded. Ideally, a large prospective cohort study should be carried out to compare women with similar risk profiles. However, this may not be feasible for studying rare outcomes, such as SAMM. To improve the quality of studies using routine registration data, these data should be improved by making recording of important risk factors, such as previous caesarean section, mandatory.

Nevertheless, we excluded major risk factors. All women in the study gave birth between 37 and 42 weeks gestation and women with induction of labour or planned caesarean section were excluded. Therefore, women with risks that required induction of labour, such as postdates pregnancy and severe pre-eclampsia or intra-uterine growth restriction and women with preterm labour were excluded. The mode of onset of labour is more accurately recorded than information on other risk factors (such as previous caesarean section) because it is compulsory to record this.

### Interpretation

It is reassuring that SAMM was not more prevalent among women that started labour in midwife-led care and, in fact, occurred less frequently.

Our results suggest that it is possible to identify a group of women at low risk of maternal complications that may benefit from midwife-led care. Although in theory it is possible that outcomes among women in midwife-led care might even have been better if they had started in obstetrician-led care, the findings are consistent with studies that compared women with similar risk profiles.[1,14] A Cochrane review showed that midwife-led continuity of care compared to other models of care is associated with better maternal and perinatal outcomes and a lower rate of medical interventions and women tend to be more satisfied.[1] Moreover, in many countries, women have more choices in place of birth if they are in midwife-led compared to obstetrician-led care.[8,15,20,21] Finally, midwife-led care may reduce health care costs.[1,14]

Although low risk women receive midwife led care in the Netherlands, they do not receive continuity of care if they develop risk factors or complications. The high and increasing rate of referrals from midwife-led to obstetrician-led care during labour is a disadvantage of the current Dutch maternity care system.[22] In 2013, 63% of nulliparous and 27% of multiparous women were referred from midwife-led to obstetrician-led care during labour.[2] After referral, care is handed over to secondary care professionals and primary care midwives no longer have an official role in the care of women. During handover, information may get lost which could potentially lead to unsafe situations.[23] In addition, women are more satisfied with the quality of their care during labour if they are assisted by their primary care midwife or a known care provider.[24] In other countries, discontinuity of care after referral is also an issue for women who start labour in midwife-led care.[25,26] An integrated care system in which primary care midwives continue to look after women with moderate risk factors and having one electronic patient file for midwife-led and obstetrician-led care may reduce some of the disadvantages of a maternity care system with a high number of referrals.[27]

The rate of medical interventions was much lower among women that started in midwife-led care compared to obstetrician-led care. These differences contributed slightly to lower risks of adverse maternal outcomes among women in midwife-led care. Augmentation of labour, operative delivery and epidural anaesthesia have all been linked to an increased risk of postpartum haemorrhage.[16,28] To weigh risks of adverse outcomes against disadvantages of medical interventions, a good risk selection and referral system is required. In several countries, obstetric indications lists are used in the decision-making process regarding consultation and referral.[3,29–31] Some are issued by midwifery organisations, others by national bodies. National risk selection guidelines should ideally be developed jointly by the main professional groups involved in intrapartum care and need to be reviewed regularly. In addition, multidisciplinary audit is essential for all cases of severe maternal morbidity so that professionals can learn from substandard care and improve collaboration.[32]

## Conclusion

In conclusion, our study showed a lower risk of severe acute maternal morbidity, postpartum haemorrhage and manual removal of placenta among women in midwife-led care at the onset of labour compared to those in obstetrician-led care. Routine registration systems in maternity care should be improved to enhance the quality of future studies using registration data. Although risk profiles differed between both groups, our results suggest that it is possible to identify a group of women at low risk of obstetric complications who may benefit from midwife-led care. Women can be reassured that we found no evidence that midwife-led care at the onset of labour is unsafe for women in a maternity care system with a well developed risk selection and referral system.

## Supporting Information

S1 Table Sensitivity analyses. (DOCX)
