# Supplementary material for: Severe Adverse Maternal Outcomes among Women in Midwife-Led versus Obstetrician-Led Care at the Onset of Labour in the Netherlands: A Nationwide Cohort Study
Source: PLoS One. 2015 May 11;10(5):e0126266. doi: 10.1371/journal.pone.0126266 (PMC4427485; doi:10.1371/journal.pone.0126266)
Supplement: S1 Table — (DOCX) [file pone.0126266.s001.docx]

**S1 Table. Sensitivity analyses**

|  |  |  |  | **Nulliparous women** | |  | **Multiparous women** | |
| --- | --- | --- | --- | --- | --- | --- | --- | --- |
|  |  |  | **N** | **Planned midwife-led care** | **Planned obstetrician-led care** | **N** | **Planned midwife-led care** | **Planned obstetrician-led care** |
| **Severe acute maternal morbidity** | Primary analysis | Adj OR (95% CI) | 98605 | 0.57 (0.45, 0.71) | Reference | 120,558 | 0.47 (0.36, 0.62) | Reference |
|  | ‘Certain’ start labour | Adj OR (95% CI) | 87849 | 0.51 (0.40, 0.65) | Reference | 115,497 | 0.42 (0.32, 0.56) | Reference |
|  | Start labour database-1 | Adj OR (95% CI) | 98829 | 0.54 (0.43, 0.67) | Reference | 120,749 | 0.53 (0.40, 0.69) | Reference |
|  | Without risk factors | Adj OR (95% CI) | 82681 | 0.60 (0.42, 0.85) | Reference | 105,897 | 0.47 (0.34, 0.66) | Reference |
| **Blood transfusion ≥ 4 p.c.** | Primary analysis | Adj OR (95% CI) | 98605 | 0.57 (0.45, 0.73) | Reference | 120,558 | 0.48 (0.36, 0.64) | Reference |
|  | ‘Certain’ start labour | Adj OR (95% CI) | 87849 | 0.53 (0.41, 0.68) | Reference | 115,497 | 0.43 (0.32, 0.59) | Reference |
|  | Start labour database-1 | Adj OR (95% CI) | 98829 | 0.54 (0.43, 0.69) | Reference | 120,749 | 0.53 (0.39, 0.70) | Reference |
|  | Without risk factors | Adj OR (95% CI) | 82681 | 0.60 (0.41, 0.86) | Reference | 105,897 | 0.47 (0.32, 0.67) | Reference |
| **Postpartum haemorrhage** | Primary analysis | Adj OR (95% CI) | 97329 | 0.70 (0.66, 0.75) | Reference | 118,678 | 0.48 (0.45, 0.52) | Reference |
|  | ‘Certain’ start labour | Adj OR (95% CI) | 86616 | 0.71 (0.66, 0.76) | Reference | 113,644 | 0.47 (0.44, 0.51) | Reference |
|  | Start labour database-1 | Adj OR (95% CI) | 97550 | 0.75 (0.71, 0.81) | Reference | 118,867 | 0.52 (0.49, 0.56) | Reference |
|  | Without risk factors | Adj OR (95% CI) | 82156 | 0.74 (0.67, 0.82) | Reference | 104,958 | 0.52 (0.48, 0.57) | Reference |
| **Manual removal of placenta** | Primary analysis | Adj OR (95% CI) | 95451 | 0.52 (0.48, 0.56) | Reference | 117,852 | 0.25 (0.23, 0.27) | Reference |
|  | ‘Certain’ start labour | Adj OR (95% CI) | 85259 | 0.49 (0.46, 0.53) | Reference | 113,084 | 0.23 (0.21, 0.25) | Reference |
|  | Start labour database-1 | Adj OR (95% CI) | 95672 | 0.57 (0.53, 0.62) | Reference | 118,039 | 0.28 (0.26, 0.31) | Reference |
|  | Without risk factors | Adj OR (95% CI) | 80470 | 0.56 (0.50, 0.63) | Reference | 104,233 | 0.28 (0.25, 0.31) | Reference |

**‘Certain’ start of labour: no discrepancies in information between databases. Start labour database-1: level of care at start of labour based on perinatal database-1, Without risk factors: women excluded who were referred from primary to secondary care during pregnancy or who had a registered risk factor.**
